# General Henry S. Turrill, M.D.

**Published:** 1907-08

**Authors:** 


### OBITUARY.

General Henry S. Turrill, M.D., of New Milford, Conn., died early in June at the offices of the Grafton Press, New York, while dictating to a stenographer material for a book relating to the bicentennial celebration to be held at New Milford, June 15-18, 1907.

General Turrill was born in New Milford, Conn., September 8, 1842. He was graduated from the Medical School of Yale University in 1864 and was at once appointed assistant surgeon in the 17th Connecticut Infantry. Honorably mustered out of the volunteer service in July of the following year, he engaged in private practice, but ten years later he was appointed an assistant surgeon in the regular army, with rank of first lieutenant. He was advanced to the rank of captain in 1880, and was promoted to surgeon, with the rank of major, in 1893.

During the war with Spain Major Turrill was appointed chief surgeon of volunteers with rank of lieutenant colonel. In 1902 he was made a deputy surgeon general in the regular army with the same rank. On March 28, 1906, he was appointed brigadier general and was placed on the retired list the next day.

General Turrill was a companion of New York Commandery of the Loyal Legion, of the United States; a member of the Army and Navy Club, of New York; the Society of the Department of the South, Military Service Institute of the United States, Sons of the Revolution and Order of Founders and Patriots of America. He was also a member of the American Public Health Association and a fellow of the Yale Medical Alumni Association. His wife, a brother and two daughters survive him.

